# The Lewy Body Dementia Domain Rating Scale: an update

**DOI:** 10.1002/alz70859_102206

**Published:** 2025-12-25

**Authors:** Joe PM Kane, Brad F. Boeve, Elie Matar, Federico Rodriguez‐Porcel, John‐Paul Taylor, The Lewy Body Dementia Domain Rating Scale steering group

**Affiliations:** ^1^ Queen's University Belfast, Belfast, Northern Ireland United Kingdom; ^2^ Department of Neurology, Mayo Clinic, Rochester, MN USA; ^3^ ForeFront Parkinson’s Disease Research Clinic, Brain and Mind Centre, University of Sydney, Sydney, NSW Australia; ^4^ Medical University of South Carolina, Charleston, SC USA; ^5^ Translational and Clinical Research Institute, Newcastle University, Newcastle upon Tyne United Kingdom

## Abstract

**Background:**

Trials in Lewy body dementia (LBD) rely on scales and measurements created for Alzheimer’s disease and Parkinson’s disease. Industry and regulatory authority stakeholders and LBD triallists have recognised the absence of an LBD‐specific scale as critical unmet need in the drug development pipeline. Heterogeneity in LBD trials design, particularly concerning outcome measures, precludes effective evidence synthesis and data harmonisation. We describe the development of the Lewy Body Dementia‐Domain Rating Scale (LBD‐DRS) and a proposed roadmap for adoption and validation.

**Method:**

Led by a core working group and steering committee, a three‐round Delphi survey process was used to establish consensus around the conceptual framework and preliminary content of the LBD‐DRS among invited LBD researchers and clinicians. “Consensus” was established when ≥75% of respondents concurred on any survey statement or questions.

**Result:**

Consensus around the need for an LBD‐specific scale, specifically for use in early LBD, was established. After feedback was received for two early versions of LBD, a third version of LBD‐DRS has been further developed. This third version, and the results of the second Delphi survey round will be presented. A pragmatic approach, favouring an inclusive scoring approach for symptoms has been adopted, recognising that further iterations will be required once field data becomes available.

**Conclusion:**

After consensus on the conceptual framework and preliminary content is established, the authors will consult with a broader range of stakeholders, including people living with LBD and their care partners, will be required to further develop LBD‐DRS. Engagement with regulatory bodies and the pharmaceutical industry will guide further development before validation studies are pursued.

